# Advances in research on autophagy mechanisms in resistance to endometrial cancer treatment

**DOI:** 10.3389/fonc.2024.1364070

**Published:** 2024-03-27

**Authors:** Jingjing Ji, Xi Cheng, Rong Du, Yuanyuan Xie, Yuquan Zhang

**Affiliations:** ^1^Department of Obstetrics and Gynecology, Affiliated Hospital of Nantong University, Nantong, China; ^2^Research Central of Clinical Medicine, Affiliated Hospital of Nantong University, Medical School of Nantong University, Nantong, China

**Keywords:** autophagy, endometrial cancer, drug resistance, chemotherapy, endocrine therapy

## Abstract

Administering medication is a crucial strategy in improving the prognosis for advanced endometrial cancer. However, the rise of drug resistance often leads to the resurgence of cancer or less-than-ideal treatment outcomes. Prior studies have shown that autophagy plays a dual role in the development and progression of endometrial cancer, closely associated with drug resistance. As a result, concentrating on autophagy and its combination with medical treatments might be a novel approach to improve the prognosis for endometrial cancer. This study explores the impact of autophagy on drug resistance in endometrial cancer, investigates its core mechanisms, and scrutinizes relevant treatments aimed at autophagy, aiming to illuminate the issue of treatment resistance in advanced endometrial cancer.

## 1 Background

The term Endometrial carcinoma (EC) denotes a type of cancerous growths found within the endometrial epithelium. Over a span of two years, the death rate for individuals with advanced EC may escalate to as much as 50%. Treating with medication plays a crucial role in improving the prognosis for advanced EC (1). However, the rise of drug resistance often leads to the resurgence of cancer or less-than-ideal treatment outcomes, creating significant difficulties in clinical environments for patients with advanced EC (2). Cellular autophagy is a process that naturally takes place and is regulated for self-repair. The method decomposes excess or slightly defective cellular components, providing vital nutrients and energy for cell survival (3). Previous studies have revealed a significant correlation between inconsistent autophagy mechanisms and tumor resistance. Changes in the autophagy mechanism could affect the reaction of cancer cells to medical interventions (4). As a result, concentrating on autophagy in conjunction with drug treatment has emerged as an innovative research field in the progression of EC. This paper delves into the connection and mechanisms connecting cellular autophagy with drug resistance in EC, aiming to illuminate this field of research.

## 2 Autophagy and endometrial cancer

### 2.1 Theoretical elements of autophagy

#### 2.1.1 The idea and categorization of autophagy

**Figure 1:**
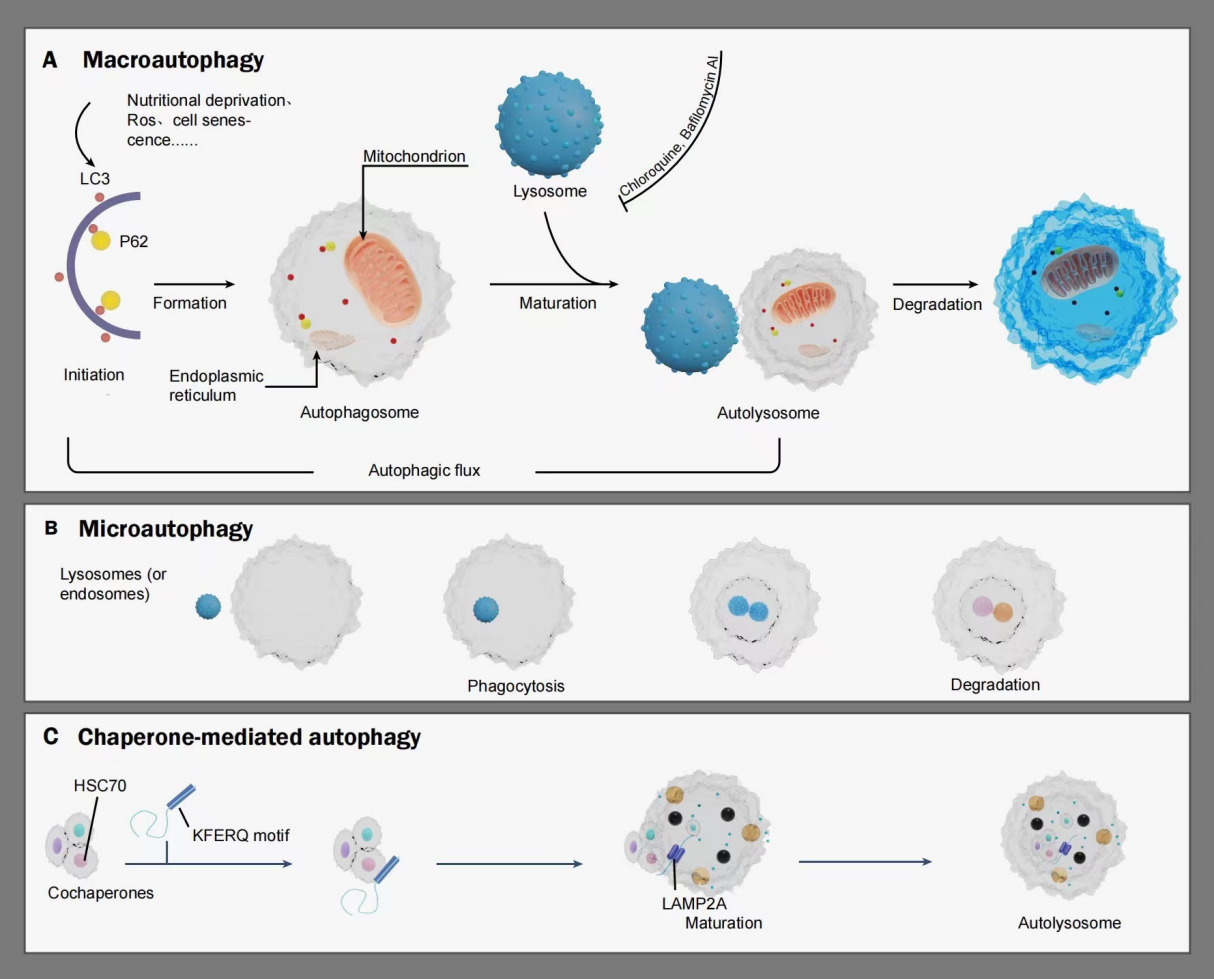
A schematic illustrating the mechanism of autophagy: Autophagy is categorized into macroautophagy, microautophagy, and chaperone-mediated autophagy. Macroautophagy is initiated under stimuli such as nutritional deprivation, reactive oxygen species (ROS), and cell senescence, gradually extending to form double-membraned autophagosomes. Autophagy markers such as LC3, P62, and organelles including the endoplasmic reticulum and mitochondrion also participate in this process. Once mature, the autophagosome fuse with lysosome to form autolysosomes, wherein lysosomal hydrolases degrade relevant cytoplasmic proteins and damaged organelles. Autophagic flux refers to the intensity of the autophagic process occurring within a certain time frame. Microautophagy involves the direct engulfment and degradation of substrates by lysosomes or late endosomes through smaller autophagic structures. Chaperone-mediated autophagy is characterized by the recognition of soluble cargo proteins containing a KFERQ sequence by the molecular chaperone HSC70. The chaperone-substrate complex binds to the lysosomal membrane receptor LAMP-2a, gradually maturing to form autolysosomes, with HSC70 mediating the translocation and degradation of substrates within the lysosomal lumen. LC3, MAP1LC3. P62, also referred to as SQSTM1, sequestosome-1. HSc70, heat shock cognate protein. KFERQ motif, a motif for a pentapeptide gene sequence. LAMP2A, lysosome-bound membrane protein 2A.

Autophagy serves as a crucial cellular process to fulfill metabolic needs and preserve cell balance by aiding in the cycling of cellular elements (5). This procedure is managed by eukaryotic cells, governed by genes associated with autophagy. This procedure is realized by decomposing its cytoplasmic proteins and impaired organelles like mitochondria, endoplasmic reticulum, and cell nucleus, with lysosomes aiding in this process. Autophagy-related (ATG) proteins comprise various ATG proteins and their core complexes, such as the ULK1/2 kinase core complex, autophagy-specific class III PI3K complex, ATG9A transport system, ATG12, and LC3 ubiquitin-like conjugation system. These proteins confer multiple activities to the autophagic pathway and participate in processes including initiation, nucleation, elongation, maturation, fusion, and degradation, all of which are crucial in cancer development (6, 7). Autophagy can be classified into basal autophagy and induced autophagy. Basal autophagy serves as a cellular self-protective mechanism essential for maintaining cellular homeostasis, synthesizing, degrading cellular products, and recycling (8). Conversely, induced autophagy promotes the survival of tumor cells during cancer progression (9), yet excessive autophagy activation can lead to metabolic stress, degradation of cellular components, and type II programmed cell death (10). Autophagy initiation can be affected by a range of internal and external factors, including lack of nutrients, reactive oxygen species (ROS), and cellular senescence. Such triggers may activate autophagy, leading to the recycling of impaired organelles like mitochondria and reducing oxidative stress within protein clusters (11).The pre-autophagosomal structure (PAS), pivotal in attracting ATG proteins, is key to triggering autophagy (12, 13). In the process of autophagy initiation, the ULK1/Atg1 functional unit, encompassing ULK1, ATG13, FIP200, and ATG101, is crucial as the starting complex for autophagy. Within this structure, ATG13 plays a crucial role in the PAS positioning of ULK1 (known as Atg1 in yeast) and in the interplay of FIP200 and ULK1. Furthermore, FIP200, known as Atg11 and Atg17 in yeast, acts as a framework for constructing subsequent ATG proteins in PAS. Following the localization of ATG13 and ULK1 to the PAS, each of these ATG proteins will initially attach to and settle on the PAS, marking the commencement of autophagy (12, 14, 15). Following this, additional functional entities such as the ULK1 complex, PI3K complex, ATG9A system, ATG12 conjugation system, and LC3 conjugation system, are systematically directed towards the PAS, contributing to the assembly and creation of autophagosomes (16–19).Autophagy is categorized into macroautophagy, microautophagy, and chaperone-mediated autophagy, depending on the various routes used to transport cell material to lysosomes. The primary route of autophagy, macroautophagy, entails trapping cell substances in double-membrane vesicles called autophagosomes, which subsequently merge with lysosomes to create autolysosomes. As implied by its name, microautophagy is the process where lysosomes or late endosomes directly consume and break down substances. During the molecular chaperone-mediated autophagy process, soluble cytosolic proteins containing the KFERQ motif are recognized by the molecular chaperone heat shock cognate protein HSC70. Subsequently, the chaperone-substrate complex binds to lysosome-associated membrane protein 2A (LAMP2A), leading to substrate unfolding. HSC70 mediates substrate translocation across the lysosomal membrane, where the substrate undergoes degradation into its constituent components by the action of hydrolytic enzymes within the lysosomal lumen, allowing cellular recycling (20). Autophagy’s key roles encompass preserving cell balance, participating in activities like anti-aging, growth and differentiation, immune response, microbial elimination, and the development of diseases, cancer included (21). (See **Figure 1**).

#### 2.1.2 Monitoring autophagy in autophagic flux

Autophagic flux denotes the total dynamic alterations happening throughout autophagy, and the build-up of autophagosomes doesn’t consistently signal the occurrence of autophagy. Given that lysosome intake may result in autophagosome build-up, assessing the autophagic flow with and without lysosomal inhibitors like bafilomycin A1 or chloroquine is crucial to differentiate these two occurrences. Consequently, overseeing autophagic flow presents a complex challenge (22). Presently, the build-up of autophagosomes is regarded as the benchmark for evaluating autophagic behavior. Biochemical tests and microscopic examination can reveal autophagosomes. The process of biochemical identification utilizes methods like immunoblotting, immunocytochemistry, and the fluorescent GFP-LC3 (23) gene to identify autophagic indicators, including microtubule-associated protein 1 light chain 3 beta (MAP1LC3B, LC3B), sequestosome 1 (SQSTM1), also referred to as p62, and beclin 1 (BECN1), proteins linked to the autophagosomal membrane. Nonetheless, such techniques typically necessitate pre-transfection. Furthermore, electron microscopy can be employed to directly detect autophagosomes.

### 2.2 Endometrial carcinoma

**Figure 2:**
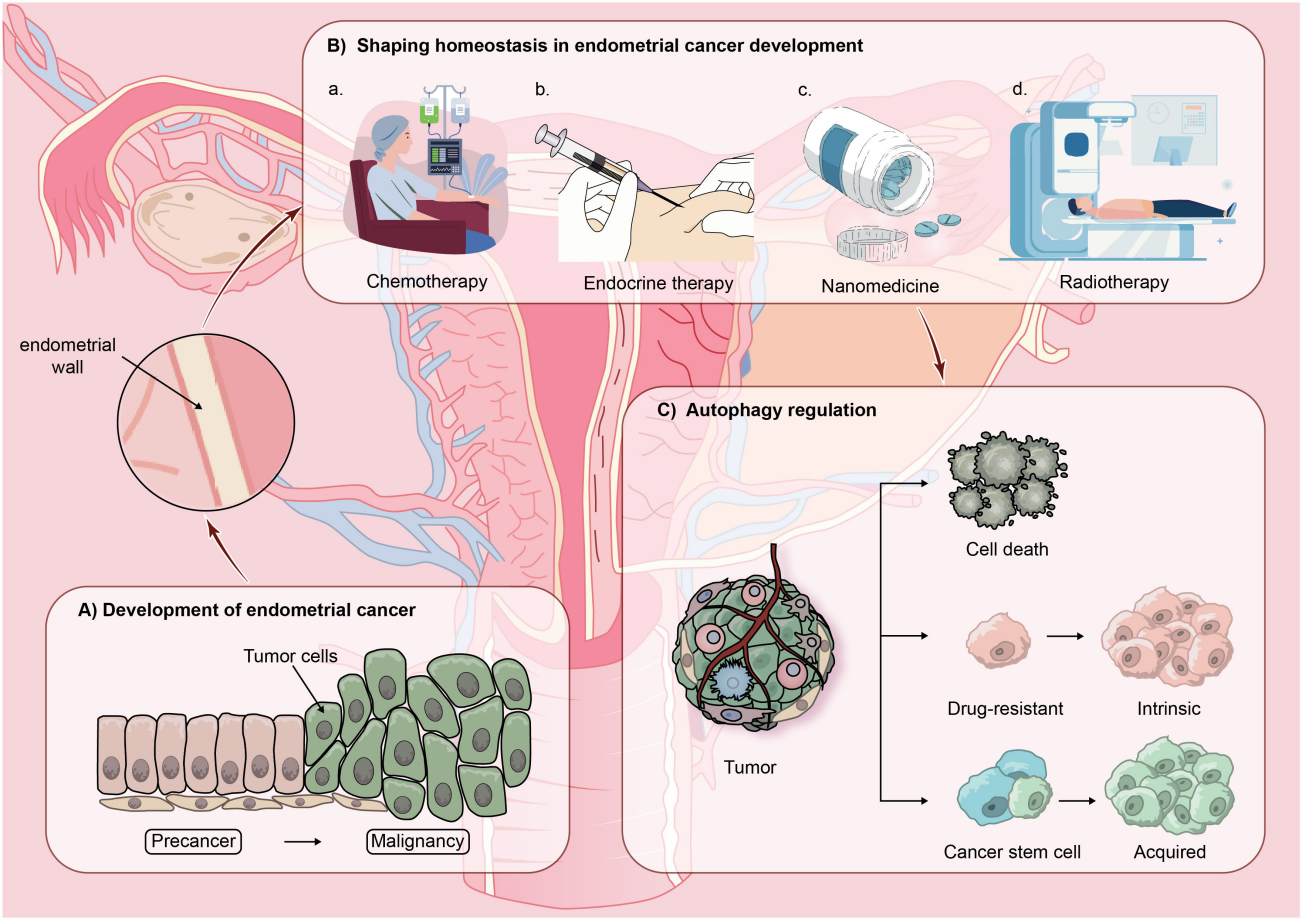
The hypothetical model proposing the involvement of autophagy in advanced recurrence of endometrial cancer (EC): EC malignant typically develops from precancerous lesions and in the progression of EC, homeostasis is gradually established in the body through treatment modalities such as chemotherapy, endocrine therapy, nanomedicine therapy, and radiotherapy. However, by modulating autophagic activity within the EC environment, three outcomes commonly emerge: cell death, cell resistance, or the continued proliferation and differentiation of residual cancer stem cells, leading to cancer recurrence.

There are two categories of EC: type I and type II. The primary category of Type I encompasses low-grade endometrioid carcinoma, typically arising from endometrial hyperplasia, triggered by overstimulation of estrogen without opposition. Type II EC encompasses advanced endometrioid carcinoma, mucinous carcinoma, and transparent cell carcinoma. Generally, these tumors do not depend on estrogen due to the absence of estrogen and progesterone receptors. In contrast to type I tumors, type II tumors tend to be more malignant. In early-stage EC patients, surgery to remove the uterus is frequently the favored treatment. In cases of patients in advanced stages, post-debulking surgery, medication is commonly used to eradicate residual cancer cells. However, the clinical results for patients with advanced EC frequently fall short of expectations. Resistance to drugs is regarded as a crucial molecular process (4). Research has shown that the diversity within tumors could play a role in enhancing drug responsiveness and resistance among cancerous cells. The diversity of tumors includes various cellular processes such as genetic alterations, epigenetic control, survival mechanisms, metabolic changes, drug removal, and the renewal of cancer stem cells, all targeting resistance and adaptation to drug-triggered cytotoxicity (24, 25). Post-medication, there are typically three outcomes: the demise of drug-sensitive cells, ongoing growth of drug-resistant cells, or the expansion and diversification of remaining cancer stem cells, culminating in cancer recurrence. (See **Figure 2**).

### 2.3 The twofold function of autophagy in endometrial cancer

In the formation of EC, autophagy serves a twofold function (26). From one perspective, enhanced autophagy may reduce the incidence of type I endometrial cancer (27). Lebovitz and colleagues Discovered notable alterations in genes associated with autophagy, namely ATG4C, RB1CC1/FIP200, and ULK4, in cases of endometrial cancer. Within this group, RB1CC1/FIP200 and ULK4 play crucial roles in starting autophagy, while ATG4C contributes to the expansion of phagocytic vesicles. Such genetic alterations could result in a reduction of autophagy, with all endometrial cancers having mutations in genes associated with autophagy classified as type I (28), autophagy’s role in inhibiting tumors in type I endometrial cancer development. Additionally, given the efficacy of calorie restriction (CR) as a stimulant in diverse metabolic tissues (29), Yin colleagues. discovered that in mice suffering from endometriosis, CR triggered a rise in autophagy rates and markedly reduced the formation of endometrial lesions (30). Given that type I endometrial cancer originates from initial lesions leading to endometrial hyperplasia, one can deduce that increased autophagy plays a role in averting the development of type I endometrial cancer. Conversely, in the case of Type II EC, Sivridis and colleagues observed a notable rise in the number of stone-like formations under immunohistochemical staining, indicative of autophagic activity, in the group with tumors, in contrast to normal endometrial tissue, correlating with unfavorable outcomes in EC (31). Su and others. Discovered that within the EC cell line ISK, heightened levels of the cell cycle protein D1 (cyclin D1, CCND1), augmented expression of BECLIN1, ATG5, ATG7, and LC3 I/II, elevated autophagy rates, and bioinformatics studies indicated that increased autophagy plays a role in the metastasis of EC lymph nodes (32). This implies that autophagy contributes to the advancement of tumors in Type II EC. In contrast, growing research indicates that various chemotherapy medications might trigger autophagy responses in cancer cells that support survival (33, 34). Blocking autophagy could make cancer cells resistant to drugs more responsive to standard chemotherapy, enhancing their responsiveness to reduced drug levels and thus diminishing adverse effects. Chloroquine, an inhibitor of autophagy, curtails the growth of EC cells resistant to cisplatin by blocking autophagy. In contrast, EC cells resistant to cisplatin show increased sensitivity to chloroquine (35), suggesting that autophagy fosters the expansion of tumor cells in these cells. Likewise, studies suggest that blocking autophagy may lead to increased progesterone resistance in endothelial cells. Enhancing autophagy suppression in progesterone-resistant EC cells through the activation of the PI3K/AKT pathway fosters the growth of cancer cells (36). Consequently, controlling autophagy not only influences the emergence of EC but also enhances the healing impacts of various medications. Currently, the scientific community categorizes the roles of autophagy mechanisms in anti-tumor drugs into four categories: cytotoxic, cytoprotective, cytostatic and non-protective (37). Qi et al. found in experiments on nude mice that TTK knockdown or cisplatin monotherapy reduced tumor volume, while combination therapy resulted in a more significant decrease in tumor volume and weight. Immunohistochemical experiments on tumor tissues showed that TTK knockdown suppressed the expression of the tumor proliferation index Ki67 and autophagy marker LC3B, while increasing the level of the apoptosis marker caspase-3, especially when combined with cisplatin treatment. This suggests that TTK knockdown can inhibit tumor growth and increase cisplatin cytotoxicity *in vivo (* 38). Zhang et al. found that increasing cisplatin concentration could induce increased autophagic activity in ovarian cancer cells, and compared to cisplatin monotherapy, cisplatin combined with autophagy inhibitors chloroquine or 3-MA could inhibit cell proliferation and promote apoptosis in ovarian cancer cells, indicating that induced autophagy plays a cytoprotective role in cisplatin-stimulated ovarian cancer cells (39). Dou et al. found that ivermectin inhibited the growth of breast cancer cells by promoting the ubiquitination degradation of PAK1, inhibiting the Akt/mTOR pathway, reducing autophagy levels, highlighting the growth-inhibitory effect of ivermectin on breast cancer cells (40). Finnegan et al. found that inhibiting autophagy with chloroquine and bafilomycin A1 and silencing the autophagy-regulating gene ATG5 did not sensitize Fulvestrant + Palbociclib to breast tumor cells, indicating that in breast tumors, the autophagy mechanism is non-protective under the action of Fulvestrant + Palbociclib (41). In summary, different anti-tumor drugs play their respective important roles by altering autophagy mechanisms under different conditions. Hence, Autophagy is vital for regulating cell quality and energy provision, and it contributes to the progression of tumors (27). Autophagy plays a multifaceted dual role in the progression and therapy of EC.

### 2.4 Development of medication resistance in endometrial cancer

Different types of tumors have distinct etiologies, which may involve genetic mutations, environmental factors, viral infections, etc. (42–44). The progression of tumors includes growth and metastasis, with significant differences in prognosis and survival status. Advanced-stage tumors typically refer to tumors that have existed for a prolonged period, with poor treatment outcomes, continuous deterioration, or spread to other parts of the body, indicating a grim prognosis for patients. For advanced-stage tumors, comprehensive treatment strategies may include a combination of surgery, radiotherapy, chemotherapy, targeted therapy, immunotherapy, and other methods. Drug therapy is commonly used to inhibit tumor progression; however, drug resistance often occurs in late-stage tumors, primarily consisting of intrinsic and acquired resistance (45). Intrinsic resistance refers to tumor cells exhibiting resistance to certain therapeutic drugs in their initial state, which may originate from genetic variations, epigenetic changes, or the presence of specific subgroups. Acquired resistance, on the other hand, refers to tumor cells gradually developing resistance to treatment drugs during the course of therapy, usually due to mutations or adaptive changes in tumor cells. Nevertheless, the mechanisms underlying both types of resistance share common features, including genetic mutations, tumor heterogeneity, activation of cellular signaling pathways, enhanced DNA repair mechanisms, and increased drug efflux pathways (46). Although drugs that induce apoptosis play a crucial role in killing tumor cells, the development of drugs targeting apoptosis-related pathways presents certain complexities and difficulties. Therefore, increasing attention is shifting towards other types of programmed cell death, among which autophagy, as a type II programmed cell death pathway, naturally becomes a focal point in the study of drug resistance mechanisms. Tumor drug resistance is a complex process involving the interaction of multiple factors, and understanding these resistance mechanisms is essential for devising effective treatment strategies and preventing the occurrence of resistance.

#### 2.4.1 Resistance to chemotherapy in EC

Research indicates a diverse range of responsiveness in various EC cells to chemotherapy medications. Doxorubicin, cisplatin, and paclitaxel are frequently employed medications in the progression of EC chemotherapy. Resistance to chemotherapy stems from intricate processes, including enhanced DNA repair, reduced apoptosis, and the initiation of autophagy, to name a few. Earlier studies have shown that reducing homeobox A11 (HOXA11) levels increases endothelial cell resistance to cisplatin through the activation of the PTEN/serine protein kinase AKT signaling pathway (47). Reducing levels of Cyclophilin A (CypA) diminishes the growth, movement, and invasive capacities of the paclitaxel-resistant EC cell lines HEC-1-B/TAX and AN3CA/TAX, while also triggering apoptosis (48). The absence of Ring finger protein 8 (RNF8) markedly increases the responsiveness of drug-resistant EC cells to cisplatin and doxorubicin in cellular survival tests. Additionally, EC cells resistant to chemotherapy demonstrate enhanced efficiency in non-homologous end joining (NHEJ) and extended preservation of the Ku80 DNA damage site on DNA double-strand breaks (DSBs) (49). By triggering the PI3K pathway, Cyclin A2 prevents apoptosis caused by cisplatin and imparts cisplatin resistance by increasing the levels of the Akt-binding protein periplakin in EC (50). Consequently, the interaction between repairing DNA damage and inducing apoptosis is vital in the resistance to EC chemotherapy. Furthermore, altering autophagy processes could enhance the responsiveness of EC cells to chemotherapy medications. Xiao and colleagues discovered that reducing levels of 6-phosphofructo-2-kinase/fructose-2,6-bisphosphatase3(PFKFB3) markedly enhanced the autophagy process triggered by carboplatin/cisplatin in EC cells HEC-1B and ARK-2, resulting in decreased cell growth and heightened responsiveness to carboplatin or cisplatin through autophagy (51). Fukuda and colleagues the study revealed that suppressing autophagy through the reduction of genes related to autophagy, namely ATG5 or ATG7, curtailed the growth of EC cells resistant to cisplatin. This reinforces the notion that autophagy inhibition can heighten the responsiveness of EC cells to chemotherapy using cisplatin, thus boosting the efficacy of the treatment (35). Liu and colleagues Discovered an elevation in autophagy in JEC and HEC-1A EC cell lines stimulated by paclitaxel (notably less responsive to paclitaxel), and showed that chloroquine’s autophagy inhibition could heighten apoptosis induced by paclitaxel (52). Consequently, the varied impacts of autophagy are crucial in the emergence of chemotherapy resistance in EC and its management, yet the precise processes are still unclear and necessitate more comprehensive investigation.

#### 2.4.2 Resistance to drug-induced endocrine drugs in EC

Adenocarcinoma, also known as endometrial hyperplasia, stems from the glandular cells in the endometrium and is closely linked to extended periods of unopposed estrogen activity. On the other hand, progestins counteract EC and facilitate regression. Consequently, hormone therapy serves as a substitute therapeutic approach, particularly for young women seeking to maintain fertility or those who cannot receive surgery, or for individuals suffering from advanced, recurring illnesses and severe complications. In the realm of endocrine treatment for EC, hormones are vital because they attach to progesterone receptors, triggering autophagy and suppressing cell growth. Nonetheless, around 30% of these patients exhibit resistance to hormone treatment (53). Research indicates that with the increasing invasiveness of tumors, the effectiveness of progestin treatment diminishes, leading to a gradual development of progestin resistance in EC (54). Nonetheless, the underlying processes of endocrine drug resistance remain largely elusive, making it crucial to delve into the molecular dynamics of progestin resistance and devise strategies to enhance the outlook for EC patients with progestin resistance. Research indicates that in endothelial cells, ABX-1431 (an inhibitor of MGLL) and Fatostatin (a derivative of dithiophene) prevent the emergence of EC and reverse resistance to progesterone by targeting the extracellular signal-regulated kinase (ERK) pathway via monoacylglycerol lipase (MGLL) and cannabinoid receptor 1 (CNR1), and also by obstructing the sterol regulatory element-binding protein 1-nuclear factor-kB pathway (55–57). Furthermore, when exposed to progestins, Brusatol, an NRF2 inhibitor, suppresses the nuclear factor erythroid 2-related factor 2-ten-eleven translocation-aldo-keto reductase family 1 member C1 (NRF2-TET1-AKR1C1) pathway, resulting in the vulnerability of endometrial hyperplasia and endothelial cells to progestins (58). When mitogen-inducible gene 6 (Mig-6) is overexpressed, it initiates an antitumor response and diminishes progesterone resistance in EC cells (59). The enzyme 3β-hydroxysterol-Δ24 reductase (DHCR24) escalates with insulin induction, enhancing the invasive capacity and progesterone resilience of EC cells (60). Additionally, an interplay exists between autophagy and endocrine resistance. The mTOR inhibitor RAD001 triggers autophagy through its interaction with the PI3K/AKT/mTOR signaling pathway, which suppresses the growth of progestin-resistant EC cells and heightens their responsiveness to progestins (36). Estrogen inhibitors are also among the medications employed in endocrine treatment. Earlier studies in clinical, biological, and epidemiological fields have indicated that prolonged or excessive estrogen exposure elevates the likelihood of EC, particularly in endometrioid tissues (61). Gu and others. Discovered that the simultaneous stimulation of phenoxodiol (PPD) and dimethylbiguanide, in contrast to estrogen-reliant EC cells, markedly decreased estrogen receptor alpha (ERα) levels in Ishikawa and RL95-2 cells. This led to a rise in BECLIN1, LC3B II/I ratio, and a reduction in P62, enhancing autophagy and eventually facilitating apoptosis (62). This indicates that the enhanced autophagy triggered by dimethylbiguanide and PPD may lessen the reliance of EC cells on estrogen, thereby elevating the rate of apoptosis in cancer cells (63). Furthermore, a method exists for integrating estrogen inhibitors into autophagy therapy. The employment of CB-839, a glutaminase inhibitor, to suppress estrogen’s function in EC cells, activates autophagy, resulting in reduced cancer cell growth and increased apoptosis in estrogen-dependent EC cells (64). To sum up, there’s a strong link between hormone-related endocrine treatment and resistance to medication in advanced-stage EC. Consequently, comprehending the underlying processes of endocrine resistance is vital to enhance the responsiveness of EC cells to hormonal medications.

#### 2.4.3 The generation of drug resistance by EC stem cells

A minor group of cancerous tumor cells, known as Cancer stem cells (CSCs), are identified as tumor-initiating cells and exhibit traits like self-renewal, metastasis, and resistance to medication. Earlier research has indicated the vital involvement of CSCs in the development of tumor resistance, dormancy, and metastasis. Consequently, focusing on CSCs serves as a potent approach to combat drug resistance in endometrial cancer (EC). When the cancer stem cell marker gene SMOC-2 is overexpressed, it triggers the Wnt signaling pathway, encourages endothelial cell growth, and heightens resistance to platinum and paclitaxel chemotherapy (65). In the realm of anti-cancer stem cell treatment, targeting the Wnt pathway is a primary focus. Furthermore, studies using flow cytometry have shown that in EC cell lines RL95-2 and RL95-2/Oct-4, heightened levels of octamer-binding transcription factor 4 (Oct-4) resulted in a doubling of cancer stem cell markers CD133 and aldehyde dehydrogenase 1 (ALDH1). The cells demonstrated enhanced growth and cloning capabilities, suggesting Oct-4’s role in triggering traits similar to endometrial cancer stem cells and boosting their resistance to platinum-based chemotherapy (66). Additionally, the suppression of ALDH activity in endometrial cancer stem cells led to a diminished reliance on the glycolytic pathway, which in turn lessened the stemness of spheroid model cells and lowered their resistance to paclitaxel (67). The heightened expression of the innate cell surface complement inhibitor CD55 in non-CSCs markedly boosted the levels of CSC markers and heightened the resistance to platinum-based chemotherapy in EC (68). Ran and colleagues Discovered that, in contrast to solely employing paclitaxel for ECSC induction, using autophagy inhibitors 3-MA or CQ together lessened ECSC proliferation and improved paclitaxel treatment responsiveness (69). To sum up, CSCs are crucial in resisting treatments and serve as essential targets for combating drug resistance in EC.

#### 2.4.4 Resistance to drugs in EC for various other treatments

Extensive studies into the development and signaling routes of EC have pinpointed specific altered genes and atypical pathways as crucial in treating EC. Consequently, the focus on targeted therapy has emerged as a prominent subject in contemporary research. Specific therapeutic medications obstruct the signaling routes or altered proteins essential for the proliferation and survival of cancer cells, thus hindering their growth. Menderes and colleagues. disclosed promising outcomes from merging anti-human epidermal growth factor receptor 2 (HER2) focused treatment with chemotherapy for individuals with advanced and recurring uterine serous carcinoma (70). Wu and colleagues Discovered that in EC, blocking the mitogen-activated protein kinase (MAPK) pathway significantly diminishes the resistance of A-type Eph receptor 2 (EphA2) to specific treatments (71). Furthermore, clinical research can focus on additional molecules, including ubiquitin-specific protease 14 (USP14), receptor tyrosine kinase-like orphan receptor 1 (ROR1), among others (72, 73). Additional factors contributing to EC resistance encompass nanoparticle treatment, which encourages the death of EC cells through the suppression of PTEN-triggered potential kinase 1/PARKIN (PINK1/PARKIN)-driven mitochondrial autophagy, thereby boosting the healing effectiveness of nano-catalysts in EC cells (74). To summarize, EC cells are capable of developing drug resistance via diverse pathways, influencing the prognosis and survival of patients. Consequently, thorough investigation into the processes behind EC resistance is essential.

## 3 The significance of autophagy in resistance to drugs in endometrial cancer

Autophagy plays a crucial role in cellular equilibrium through the breakdown and reuse of cellular elements. The control and functioning of autophagy in endometrial cancer (EC) are shaped by pathways related to autophagy, genetic factors, and levels of protein expression. Given autophagy’s crucial function in the development of EC and resistance to drugs, focusing on molecules or signaling pathways related to autophagy is seen as a crucial advancement in improving the effectiveness of drug therapies in EC.

### 3.1 PI3K/AKT/mTOR

The PI3K/AKT/mTOR pathway stands as a traditional signaling route in the process of cellular autophagy. PI3K, a heterodimer, consists of the regulatory unit p85 and the catalytic unit p110. Upon attaching to growth factor receptors like EGFR, it modifies AKT’s protein architecture and activation condition, thus either stimulating or suppressing various downstream elements, including cell apoptosis-related proteins like Bad and Caspase9, which in turn control cell growth, differentiation, apoptosis, movement, and other biological activities. Furthermore, AKT is capable of engaging with the kappa B kinase (IKK) inhibitor and the nuclear factor-k-gene binding (NF-κB) pathway. The PI3K/AKT signaling pathway primarily targets mTOR, with its subsequent transcription factors encompassing hypoxia-inducible factor (HIF1α), oncogenes like c-MYC in the Myc gene family, and forkhead box O (FOXO), among others. A multitude of research has shown that blocking the PI3K/AKT/mTOR pathway may lead to heightened cellular autophagy processes. Within the EC cell lines Ishikawa and HEC1-B, escalating levels of ginkgolic acid progressively suppressed the PI3K/AKT/mTOR pathway, boosting autophagy in cancerous cells, thus curtailing cell growth and triggering apoptosis (75). Inhibiting the PI3K/AKT/mTOR pathway in EC cell lines HEC-1A and Ishikawa led to increased autophagy activity and reduced cancer cell proliferation, migration, and invasion, achieved by inhibiting FAM83B, a Family 83 member with similar sequences. On the other hand, the suppression of FAM83B following the activation of the PI3K/AKT/mTOR pathway led to a decrease in autophagy in EC cells, yet enhanced the growth, movement, and invasive capabilities of cancer cells (76).In breast cancer cell lines, elevated expression of FAM83B activates the PI3K/AKT signaling pathway (77) and stimulates mTOR activation (78). Increasing evidence suggests that targeting the PI3K/AKT/mTOR signaling pathway to regulate autophagy activity has become an important therapeutic strategy for various tumors, playing a crucial role in enhancing the chemosensitivity of tumor cells and avoiding drug resistance (79). Therefore, fluctuations in the expression levels of FAM83B molecules constantly influence the progression of autophagy. Plumbagin (PLB), primarily found in the plant kingdom, belongs to the class of secondary metabolites and is mainly extracted from the roots of the Plumbago genus (80, 81). PLB induces cell apoptosis and cell cycle arrest by generating intracellular reactive oxygen species, exerting anti-proliferative effects in various tumor cells (82, 83). PLB boosts autophagy by blocking the PI3K/AKT pathway, which results in the initiation of apoptosis and the prevention of cell invasion in Ishikawa cells (84). To sum up, focusing on the PI3K/AKT/mTOR signaling route to control autophagy function can somewhat prevent the emergence of EC drug resistance and increase responsiveness to medicinal therapies.

### 3.2 ATG5/BECLIN1

The proteins ATG5 and BECLIN1 are crucial in creating autophagosomes, significantly contributing to the autophagy process. Not only do they encourage the creation of autophagosomes, but they also trigger cell death, acting as key molecular regulators of both cell autophagy and apoptosis. ATG5 is involved in constructing the ubiquitin-like conjugation system, encompassing the ATG5-ATG12-ATG16L complex, crucial for the creation of autophagosomes. Conversely, BECLIN1 plays a role in forming the phosphatidylinositol 3-kinase (PtdIns3KC3) complex, a process that triggers cellular autophagy. The natural diterpene Sugiol mainly originates from the Danshen genus, juniper, and dawn redwood (85, 86). This entity exhibits a broad range of biological functions, including antioxidant, antibacterial, anticancer, and anti-inflammatory properties (86). Sugiol is capable of triggering cell death to halt the growth of human ovarian cancer cells (87) and can also halt the cell cycle at G2/M, thereby preventing pancreatic cancer cells from proliferating (88). Earlier research indicates that both ATG5’s N-terminal truncation molecule (tATG5-N) and BECLIN1’s C-terminal truncation molecule (BECLIN1-C) are capable of triggering cell apoptosis. Administering Sugiol to EC cells HEC-1-B resulted in elevated levels of LC3B-II, BECLIN1, ATG5, and ATG12, but a reduction in P62 expression. The findings verified a notable increase in autophagy triggered by Sugiol, resulting in the suppression of EC cell growth and indicating Sugiol’s potential to curb cancer cell proliferation in EC through autophagy induction (89). Artemisinin derivative artesunate (ART) is a well-modified derivative of artemisinin, whose effects are not limited to fever, hemorrhoids, and malaria (90), but also exhibit anticancer properties in various cancers such as cervical cancer, breast cancer, ovarian cancer, and colorectal cancer (91–93).Additionally, literature suggests that ART can influence multiple processes in cancer cells, including inhibiting angiogenesis, proliferation, cell cycle arrest, ferroptosis, etc (94–96). Recent studies have found that ART can also exert anticancer effects by regulating autophagy in tumor cells (95). Zhang et al. discovered that ART not only actively hinders the growth and movement of EC cells, encourages cell death, but also increases the presence of the co-stimulatory molecule CD155 in EC cells by triggering ATG5-related autophagy. Consequently, this amplifies the harmful effects of natural killer 226 cells via the interplay of CD92, CD155, and the TIGIT co-inhibitory receptor, which in turn facilitates the cancer-fighting capabilities of EC cells and produces twofold anti-cancer impacts (97). Furthermore, Liu and colleagues discovered that diminishing BECLIN1 activity diminishes autophagy, amplifies apoptosis in EC cells triggered by paclitaxel, and enhances paclitaxel’s efficacy in chemotherapy (52). Sun and others. It was found that EC cells resistant to Ishikawa platinum demonstrate greater autophagy rates than their platinum-sensitive counterparts. The comparative expression study of long non-coding RNA (lncRNA) showed a notable decrease in HOX transcript antisense RNA (HOTAIR) levels in cells resistant to treatment. Additionally, it was discovered that HOTAIR has the ability to control BECLIN1 expression. Reducing HOTAIR levels through siRNA amplifies the autophagic activity in Ishikawa cells resistant to platinum, thereby advancing apoptosis and boosting EC cells’ resistance to platinum-based medications (98). Generally, ATG5 or BECLIN1 could play a crucial role in the emergence of chemoresistance within EC.

### 3.3 HMGB1

HMGB1, a protein found extensively in the nucleus and cytoplasm, has the capability to be secreted into the extracellular space. HMGB1 in the nucleus attaches to DNA and RNA, which in turn controls the stability of the genome and the transcription mechanisms. The cytoplasmic HMGB1 is involved in controlling autophagy through its interaction with fundamental autophagy regulatory elements. HMGB1, once secreted, serves as a binding agent for multiple receptors like RAGE and TLR, influencing several signaling routes, such as mitogen-activated protein kinase (MAPK), PI3K, and NF-κB signaling. HMGB1 is vital in regulating autophagy, with its movement aiding in the prolongation of autophagy following cellular stress (99). Furthermore, HMGB1 is capable of triggering cell growth, movement, and programmed cell death (100). Current studies demonstrate the interplay between HMGB1 and multiple elements in cancer evolution, including BECLIN1, advanced glycation end products RAGE, and PI3K, influencing the advancement of cancer (101–103). Ran and colleagues Discovered that miR-218 specifically affects HMGB1, and its heightened expression inhibits HMGB1-driven autophagy in paclitaxel-resistant EC cell lines RL95-2 and Ishikawa. Consequently, there’s a reduction in the growth ability of cancer cells, reinstating the responsiveness of resistant EC cells to paclitaxel (104). Despite the scarcity of studies on HMGB1 in EC, research indicates a strong link with its resistance to chemotherapy in cases of ovarian and cervical cancer. Zhang and colleagues It was found that HMGB1 regulates autophagy through NAC1 (ovarian cancer-associated gene 1) in ovarian cancer cells. Reducing NAC1 activity leads to lower HMGB1 levels and diminished autophagy, causing reduced growth and heightened apoptosis in ovarian cancer cells triggered by cisplatin, thereby increasing the cancer’s vulnerability to cisplatin (39). Xia and colleagues Discovered a temporal escalation in HMGB1 expression in cervical cancer cells following cisplatin treatment, coupled with heightened autophagy activity. Yet, reducing HMGB1 levels leads to a decline in autophagy, a rise in apoptosis, and a decline in cell proliferation. The findings demonstrate how HMGB1 enhances the responsiveness of cervical cancer cells to cisplatin therapy (105). Consequently, focusing treatment on HMGB1 could be a crucial approach to reinstate chemotherapy responsiveness in EC cells that have become resistant to medication.

### 3.4 MicroRNA

miRNAs, or MicroRNAs, represent a category of brief non-coding RNA strands, each spanning roughly 21-25 nucleotides. MiRNAs share a close connection with epigenetic processes. Reports indicate that miRNAs, in conjunction with various epigenetic elements, regulate EC, influencing its emergence, growth, angiogenesis, and resistance to medication. MiRNAs are capable of controlling the expression of various genes that play a role in the development of EC. Current studies indicate that miRNAs play a role in controlling tumor development and lymph node spread in living organisms, and in laboratory conditions, they participate in the invasion, movement, and epithelial-mesenchymal transition (EMT) of EC cells (106). Ran and colleagues demonstrated that suppressing HMGB1-driven autophagy through increased miR-218 expression can lessen EC cells’ resistance to paclitaxel, thereby reinstating their responsiveness to paclitaxel in resistant EC cells (104). Different research by Zhuo and colleagues revealed that in Ishikawa’s progesterone-resistant EC cell line, blocking miR-205 enhances PTEN expression and markedly reduces the levels of crucial downstream targets like phosphorylated AKT and mTOR, thus boosting autophagy. Eventually, this results in the programmed cell death of EC cells resistant to progesterone, reinstating their responsiveness to progesterone (107). Within the realm of gynecological cancer, miRNAs, alongside EC, are significantly involved in ovarian cancer (OC). Hu and others. Research has shown that reducing the expression of the Forkhead box protein P1 (FOXP1)/autophagy-related gene 14 (ATG14) pathway through the overexpression of miR-29c-3p can suppress autophagy in OC cells and increase susceptibility to cisplatin (108). Yu et al. discovered in a different research that enhancing microRNA 1301 in drug-resistant OCs can suppress the expression of genes related to autophagy, such as ATG5 and BECLIN-1, leading to decreased autophagy and fostering the growth and invasion of cancer cells. In contrast, the reduction of microRNA 1301 resulted in a contrary impact (109). Furthermore, Song and colleagues Discovered that the heightened expression of miR-219-5p, which triggers the Wnt/β-catenin pathway, might suppress HMGA2-driven autophagy and increase responsiveness to cisplatin in OC cells (110). To sum up, miRNAs control autophagy processes by focusing on various genes and signaling routes, thereby enhancing the responsiveness of cancer cells to medications. Consequently, exploring the link between miRNAs and autophagy is crucial when examining drug resistance mechanisms in EC.

### 3.5 Additional

Beyond the previously described processes controlling autophagy, additional crucial proteins or inhibitors of the autophagy pathway are involved in treating EC and significantly influence the regulation of drug resistance. Take p53 as an instance; it’s a tumor-inhibiting protein capable of modulating cell autophagy via multiple routes, mainly based on its position within the cell. Within the nucleus of a cell, p53 has the ability to enhance autophagy by stimulating specific upstream controllers of mTOR. Wu and colleagues Discovered that in endothelial cells, palmitic acid ester reduces p53 levels, leading to an increase in autophagy indicator LC3B, a rise in apoptotic indicators CHOP and PARP, fostering cell death, and heightening responsiveness to cisplatin and doxorubicin therapy (111). The mTOR inhibitor RAD001 stands as another significant inhibitor. Wang and colleagues Discovered that RAD001, in contrast to solely using paclitaxel for EC therapy, triggers autophagy by reducing AKT/mTOR phosphorylation, markedly hindering the growth of human EC cells Ishikawa. This indicates that the joint treatment involving RAD001 and paclitaxel increases the responsiveness of EC cells to paclitaxel (112). Sorafenib, a similar medication, induces reliance on an initial defensive autophagic reaction and boosts autophagy in EC cells through activating the mitogen-activated protein kinase (MAPK)/c-Jun N-terminal kinase (JNK) process. Merging sorafenib with the autophagy inhibitor CQ and subcutaneous injection into a xenograft mouse model led to a notable reduction in tumor size after two weeks. This indicates that focused autophagy inhibition can amplify sorafenib’s cytotoxic effects and suppress EC growth (113), thereby boosting sorafenib’s efficacy in EC treatment. Furthermore, in EC cells, resveratrol (RSV) triggers apoptosis in cancer cells and stimulates adenosine monophosphate-activated protein kinase (AMPK) or extracellular signal-regulated kinase (ERK), leading to the initiation of autophagy. Consequently, autophagy stimulates cell metabolism, fostering the growth of cancer cells to offset the apoptosis of EC cells caused by RSV. Nonetheless, the joint use of the autophagy inhibitor CQ and RSV markedly elevates apoptotic cancer cell counts, thereby boosting the responsiveness of EC cells to RSV therapy (114). Additionally, when exposed to the sulfated glycosaminoglycan PG545 and chemotherapy agents’ paclitaxel and cisplatin, ER stress is initiated, resulting in a heightened LC3II/I ratio and improved autophagy processes. Consequently, this escalates the apoptosis of cancer cells and heightens the responsiveness of EC cells to paclitaxel and cisplatin (115). To sum up, an in-depth comprehension of molecules involved in autophagy and their signaling routes is vital for enhancing endothelial cell responsiveness to medicinal treatments.

## 4 Feasibility of targeting autophagy in improving clinical treatment of advanced endometrial carcinoma

The diminished outlook for treatment and lower life quality in advanced EC primarily stem from the ongoing growth, diversification, and emergence of drug resistance in ECSCs, with autophagy being a key factor in these dynamics. Initially, in the realm of targeted therapies focusing on autophagy-related pathways, as mentioned above, the PI3K/AKT/mTOR signaling pathway’s prominent role in autophagy has also emerged as a clinical research hotspot for improving the prognosis of endometrial cancer. However, the development of novel therapeutic agents targeting molecules on the PI3K/AKT/mTOR pathway presents certain challenges in the implementation of clinical research and treatment. PI3K inhibitors are categorized into pan-PI3K inhibitors and subtype-selective PI3K inhibitors. Preclinical studies have shown that EC cell lines carrying mutations in PIK3CA and PTEN exhibit selective sensitivity to GDC-0941 (a pan-PI3K inhibitor) (116). LY2942002 (a PI3K inhibitor) demonstrates apoptotic effects in EC cell lines and impedes EC growth in nude mice (117). However, these compounds exhibit significant toxicity in animal studies, rendering them intolerable for prolonged periods, thus presenting challenges in targeting the PI3K family in clinical research (118). Presently, in the development of mTOR inhibitors, everolimus and temsirolimus (rapamycin derivatives) have shown anti-tumor activity in EC cell lines, with everolimus exhibiting the ability to slow endometrial hyperplasia progression in inducible Pten gene knockout mouse models (119). Temsirolimus demonstrates higher sensitivity in high-grade EC cells compared to cisplatin, doxorubicin, and paclitaxel (120). In phase II clinical studies, the combined use of everolimus and letrozole results in a high clinical benefit rate and objective response rate in recurrent EC patients, surpassing overall expectations, and emphasizes everolimus’s tolerability in pretreated EC patients (121). Considering that targeting only one mTORC complex inhibitor may induce negative feedback regulation in the PI3K/AKT/mTOR pathway, second-generation mTOR inhibitors have been developed, capable of targeting the catalytic sites of both mTOR complexes. In EC cell lines and xenograft mouse models, mTORC1/2 inhibitors AZD8055 and OSI-027 inhibit cancer cell growth (122, 123). Current research data indicate that AKT mutations are rare in EC, making AKT inhibitors a potential breakthrough in improving EC prognosis treatment. However, due to numerous feedback loops and crosstalk in the PI3K/AKT/mTOR pathway, clinical research difficulties are correspondingly increased. Similarly, the Ras/Raf/MEK/ERK (mitogen-activated protein kinase, MAPK) pathway is also involved in autophagy regulation. However, due to the difficulty in specifically targeting RAS family molecules, the development of inhibitors for this signaling pathway mainly focuses on downstream kinases RAF and MEK (124). However, currently developed RAF and MEK inhibitors as monotherapies exhibit limited therapeutic efficacy, and resistance mechanisms are poorly understood (125), indicating the need for innovative approaches to target and study this pathway.

Secondly, in targeting autophagy-related molecules, it is observed that in the prognostic analysis of EC resistance, molecules such as Sirtuin 1, P53, and PTEN are associated with resistance to different drugs and prognosis survival rates (126, 127), and these molecules are linked to autophagy. In clinical practice, these molecules can improve the prognosis of EC by inducing autophagy. Furthermore, modulating autophagy induction or inhibition can enhance the sensitivity of drugs to cells. Currently, commonly used autophagy activators include Rapamycin and Metformin (128). For example, studies have shown that Rapamycin can increase apoptosis in endometrial cancer cells induced by cisplatin, exhibiting a synergistic effect with cisplatin (129). Additionally, targeting autophagy with metformin in endometrial cancer cells resistant to progesterone has been shown to increase the expression of LC3 and BECLIN 1, promoting cell apoptosis and enhancing cell sensitivity to progesterone (130). Autophagy inhibitors include Chloroquine, Hydroxychloroquine, and 3-MA (128). Studies have demonstrated that the combination intervention of chloroquine and paclitaxel leads to decreased autophagic activity and a higher proportion of cell death in HEC-1A and JEC cells (52, 131). Furthermore, the autophagy inhibition of 3-MA results in the downregulation of L-type calcium channel α1D subunit Cav1.3 and enhances cell death induced by nitrendipine (132). Although these drugs have been proven to regulate autophagy activity, they have not yet been applied in clinical practice due to the potential for various side effects both *in vitro* and *in vivo (* 133). Among them, Rapamycin may cause side effects such as immunosuppression and oral ulcers (134), Metformin may lead to lactic acidosis (135), and the side effects of Chloroquine and Hydroxychloroquine include gastrointestinal discomfort, neurotoxicity, retinal toxicity, and cardiotoxicity (136–139). Therefore, careful evaluation of the safety and efficacy of these drugs is necessary in clinical application to achieve better therapeutic outcomes.In summary, modulation based on autophagic activity can enhance the efficacy of late-stage endometrial cancer treatment and provide new avenues for clinical treatment advancement. Thus, targeted therapy for autophagy has potential implications in improving the sensitivity of endometrial cancer treatment and may contribute to the development of clinical research related to autophagy-mediated endometrial cancer resistance.

## 5 Exploring the potential and constraints of focusing on autophagy to enhance resistance to medication in EC

The development of resistance significantly hampers the effectiveness and reliability of specific cancer treatments (140). Cells that are not resistant to mutations may act as a breeding ground for entirely resistant cells, potentially resulting in a recurrence of tumors (141–143). Changes in autophagy processes play a role in the resistance of tumor cells to different medications, as evidenced in cases of endometrial cancer, ovarian cancer, cervical cancer, and breast cancer cells (38, 53, 144). Yet, as of now, no clinical trial findings have been published regarding the enhancement of drug resistance in endometrial cancer cells via specific autophagy medications. Consequently, clinical studies focusing on specific autophagy show potential in surmounting drug resistance in EC. Typically, advanced EC treatment encompasses a mix of biomarker-based precision therapy, immunotherapy, targeted therapy, and radiochemotherapy. As new drugs surface, we aim to create tailored and ideal diagnostic and therapeutic approaches.

Investigating autophagy and drug resistance mechanisms in EC presents specific constraints. EC is categorized into two types: Type I, which relies on estrogen, and Type II, which does not. Conversely, Type II EC usually lacks estrogen receptors, is undifferentiated, and is linked to severe and unfavorable outcomes, representing 15% of all cases yet accounting for half of the recurrences, rendering Type II EC research more significant. Nonetheless, conclusively classifying EC cells as either Type I or Type II can be difficult at times, with present-day histological categorization still being the benchmark for patient layering. Although some studies employ cell lines not deemed indicative of Type II EC, the analogous gene expression patterns of easily obtainable EC cells have led to an absence of comprehensive subtyping in the majority of research. However, later molecular experimental studies have yielded encouraging outcomes, offering crucial insights for enhancing the precision of prognoses and forecasting reactions to new treatments.

## 6 Final thoughts and future outlook

Resistance to medication in advanced EC continues to be a major hurdle, highlighting the need to modify patient responsiveness to drug treatments for better prognosis. The process of autophagy is vital in the emergence and evolution of EC, where alterations in autophagic flow are intimately linked to the resistance of tumors. Specific treatments targeting autophagy have been utilized in a range of illnesses, such as cancer, neurodegenerative diseases, aging, and inflammation. Therapeutic combinations focusing on autophagy could be a hopeful approach in the fight against tumor resistance. With the advancement of studies on autophagy in endothelial cells, two distinct paths warrant investigation. Initially, autophagy represents a multifaceted network governed by multiple factors, encompassing genetic and metabolic elements. Presently, the comprehension of autophagy’s role in the mechanisms of drug resistance in EC remains incomplete. Consequently, an in-depth comprehension of the pertinent targets and distinct autophagy signaling routes in EC is essential for accurate therapy. Additionally, certain experimental medications have shown promise in controlling the autophagy of tumors. These medications require additional research and continuous investigation via preclinical and clinical trials to hasten their integration into clinical treatment methods and offer innovative strategies for surmounting therapeutic hurdles in clinical environments. To sum up, additional studies into how autophagy affects resistance to drug treatments in EC are of substantial scholarly and clinical importance. Gaining an in-depth insight into the control processes of autophagy and creating specific treatments for it will lead to more efficient therapies for EC patients, thereby enhancing their prognosis and life quality.

## Author contributions

JJ: Writing – original draft. XC: Writing – review & editing. RD: Writing – review & editing. YX: Writing – review & editing. YZ: Writing – review & editing.
